# Simulation-Based Summative Assessment of Neonatal Resuscitation Providers Using the RETAIN Serious Board Game—A Pilot Study

**DOI:** 10.3389/fped.2020.00014

**Published:** 2020-01-31

**Authors:** Simran K. Ghoman, Maria Cutumisu, Georg M. Schmölzer

**Affiliations:** ^1^Neonatal Research Unit, Centre for the Studies of Asphyxia and Resuscitation, Royal Alexandra Hospital, Edmonton, AB, Canada; ^2^Department of Pediatrics, Faculty of Medicine and Dentistry, University of Alberta, Edmonton, AB, Canada; ^3^Department of Educational Psychology, Centre for Research in Applied Measurement and Evaluation, University of Alberta, Edmonton, AB, Canada; ^4^Department of Computing Science, University of Alberta, Edmonton, AB, Canada

**Keywords:** infants, newborn, neonatal resuscitation, simulation based education, serious game, summative assessment

## Abstract

**Background:** Each year, 13–26 million newborn babies require help to breathe at birth. Healthcare professionals (HCPs) who provide neonatal resuscitative care must be frequently evaluated to maintain and improve the quality of healthcare delivered. While simulation-based competence assessment is preferred, resource constraints hinder uptake. We aimed to examine if the RETAIN simulation-based boardgame can be used to assess HCPs' neonatal resuscitation knowledge.

**Method:** Twenty neonatal HCPs (19 females) from the Royal Alexandra Hospital (Edmonton, Canada) were recruited. First, they completed an open-answer written test of one neonatal resuscitation scenario. Then, they completed one neonatal resuscitation scenario of difficulty comparable to that of the open-answer written test, but this time using the RETAIN board game. In the RETAIN board game (https://playretain.com, RETAIN Labs Medical Inc, Edmonton, Canada), players perform simulated neonatal resuscitation scenarios based on real-life cases, using action cards, and equipment pieces. Sessions were video-recorded and scored using Neonatal Resuscitation Program 2015 guidelines. Data are reported as mean (standard deviation) for normally distributed continuous variables, and as median (interquartile range) for non-normal continuous variables.

**Results:** Participants consisted of the following HCPs: 8 nurses, 4 respiratory therapists, 4 nurse practitioners, and 4 neonatal fellows with median (IQR) 10.5(3–17) years of clinical experience. Overall mean (SD) *Open-answer test* and *Game Performance* was 8.6(2.1) out of 16 possible points (53%) and 29(3.2) out of 40 possible points (74%), respectively. Out of the 10 actions shared between the open-answer test and game scenario, performance on the *open-answer test* was mean (SD) 7.2(1.3) (72%) and *game performance* was mean (SD) 8.8(1.4) (88%) (V = 17, *p* < 0.01).

**Conclusion:** RETAIN may provide an enjoyable and standardized alternative toward summative assessment of neonatal resuscitation providers. RETAIN may be used to improve more frequent and ubiquitous uptake of simulation-based competence assessment in healthcare settings.

## Introduction

Every day, patients and caregivers place their trust in the hands of healthcare professionals (HCPs) to provide high-quality care that improves health outcomes. Indeed, each day, over 35,000 newborn infants around the world will need help to take their first breaths (1). These newborn infants and their families depend on HCPs to perform complex interventions quickly and accurately, under highly-stressful conditions (2). To ensure that the quality of healthcare being delivered meets expected standards, lifelong assessment of neonatal HCPs' competence is needed (3). Therefore, guidelines recommend the biennial Neonatal Resuscitation Program (NRP) provider certification course (4). However, despite this recommendation, half of infant mortality during neonatal resuscitation is caused by deficiencies in HCPs' competence (5). More frequent and objective summative assessment of neonatal HCPs is required to address this gap.

Summative assessment evaluates a learner's individual performance using a final score that indicates their position in comparison to others or to an expected standard (6). While independent simulation-based summative assessment demonstrates HCPs' neonatal resuscitation competence, resource constraints hinder its regular use in high-stakes testing, such as during NRP certification (7, 8). Recognizing these constraints, simulation-based serious games may provide an attractive alternative (8).

Serious games create an immersive environment, using elements like competition and emotional design, to teach players relevant knowledge, or skills through problem-based learning (9). While serious games in medical education have been used for training competency (10, 11), simulation-based serious games may also provide a solution to the need for more frequent, accessible, and effective assessment of NRP-providers' competence. This study aimed to examine if the simulation-based serious board game RETAIN can be used as a summative assessment method. We hypothesized that individual performance on the serious board game RETAIN compared to performance on an open-answer written test will provide a more comprehensive summative assessment of HCPs' neonatal resuscitation competence and will uncover HCPs' deeper neonatal resuscitation learning.

## Methods

Twenty neonatal HCPs trained in NRP (e.g., registered nurses, nurse practitioners, respiratory therapists, residents, and fellows) were recruited from the Neonatal Intensive Care Unit (NICU) at the Royal Alexandra Hospital, Edmonton, Canada—a tertiary perinatal center admitting over 350 infants with a birth weight of up to 1,500 g annually. The study was performed at the simulation lab at the Center for the Studies of Asphyxia and Resuscitation, Edmonton, Canada. The study was approved by the Human Research Ethics Board at the University of Alberta, Edmonton, Canada (Pro00085274), and written informed consent was obtained from the HCPs prior to their participation.

### Recruitment

HCPs from the Resuscitation Team (i.e., experienced neonatal HCPs who regularly attend high-risk deliveries and resuscitations) at the Royal Alexandra Hospital NICU were recruited for this study based on their availability. A research nurse assisted with recruitment. Anonymized demographic information (including clinical position) was collected from participants in a pre-survey before the study. This information was continuously reviewed during recruitment to aim for a representative sample of HCPs who typically attend deliveries as the Resuscitation Team and to avoid over-representation of one group. At this site, registered nurses constitute the main group of HCPs who attend resuscitations, and are aided by respiratory therapists, doctors, and nurse practitioners.

### The RETAIN Board Game

**Figure 1:**
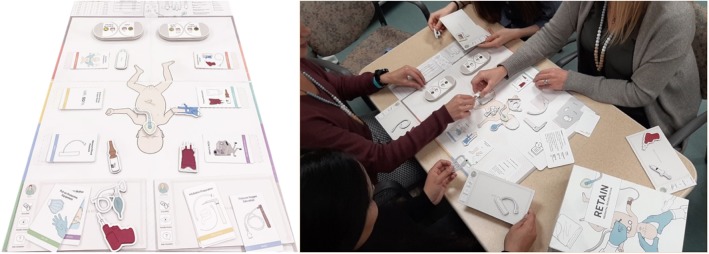
A team playing the RETAIN collaborative board game.

RETAIN (REsuscitation TrAINing for healthcare professionals) is a simulation-based serious board game (https://www.playretain.com, RETAIN Labs Medical Inc. Edmonton, Canada) for HCPs to simultaneously practice their knowledge of the neonatal resuscitation guidelines and communication skills. The collaborative table-top training simulator consists of a game board with a center-piece image of a newborn infant (Figure 1). Players take on the role of an HCP attending deliveries and use equipment pieces (i.e., T-piece), action cards (i.e., start PPV), and adjustable monitors (i.e., fraction of inspired oxygen) to perform interventions (Figure 1). A facilitator guides the scenario by providing feedback of the infant's cardiorespiratory status. To successfully stabilize the infant, players must seek and use this information to make appropriate decisions, based on the latest edition of the NRP guidelines. Moreover, after each scenario, players use debrief cards to reflect on their performance (i.e., “What did you learn?” or “What could have gone better?”). Scenarios in the RETAIN serious game are based on a clinical database of real-life resuscitations from the Royal Alexandra Hospital delivery room. They were selected to match the open-answer test (administered before the board game) in difficulty.

### Study Design

Each participant completed a demographic questionnaire (i.e., time elapsed since last NRP-recertification course) and an open-answer test to assess their neonatal resuscitation knowledge. In the open-answer test, participants were instructed to answer prompts by writing the steps they would take to resuscitate and stabilize an apneic 24-week premature infant (Appendix 1). The Royal Alexandra Hospital NICU is a tertiary perinatal center that admits <350 infants with <1,500 g birthweight annually and HCPs attending deliveries are all trained in the standard protocols of care for resuscitating premature infants between 22 and 42 weeks gestation. Participants received no feedback about their performance on the open-answer test. Next, each participant completed one scenario of the RETAIN board game for about 30 min. After receiving instructions on how to play the game, participants were informed about an imminent delivery. Using action cards and equipment pieces, participants independently prepared for and performed the resuscitation of an infant with fetal bradycardia (Appendix 2). The scenario was moderated by a facilitator (SKG) who reported the infant's heart rate, oxygen saturation, work of breathing, and visual appearance throughout the scenario as feedback available to the players during the game, but provided no assistance or help. While the open-answer test and the game scenario had different answer keys, the two scenarios were of comparable difficulty.

Afterwards, participants completed a post-game questionnaire that collected information about their board game habits (e.g., “How many hours do you spend playing board games in a typical month?”) and mindset (e.g., “How much do you agree with the following statement? You can always change how good you are at your job”).

Sessions were audio- and video-recorded using a GoPro camera (GoPro, Inc., San Mateo, CA) and they were blindly scored by a trained evaluator, which was a laborious and lengthy process. Open-answer test and game performance were scored using 7th edition NRP guidelines.

### Measures

The *Open-answer test* measure represents the cumulative score across all actions, interventions, or tasks described by the participant on the open-answer written test scenario. The maximum score for each participant, when answering all actions, interventions, and tasks correctly, was 16 points, with a range from 0 to 16. The *Game Performance* measure represents the cumulative score across all actions, interventions, or tasks described by the participant in the board game scenario. The maximum score for each participant, when answering all actions, interventions, and tasks correctly, was 40 points, with a range from 0 to 40. Participants were assigned one point for each correct action, intervention, or task on both measures. The Open-answer test and Game Performance measures shared 11 common actions, interventions, or tasks across both scenarios (Appendix 3).

The *Years of Neonatal Experience* measure represents the participant's number of years of clinical neonatal experience. The *Years of Board Game* measure represents the participant's number of years playing board games. The *Enjoyment* measure represents the participants' self-reported enjoyment of playing the board game on a 5-point Likert scale (1 = Strongly Disagree to 5 = Strongly Agree).

Data are presented as median [interquartile range (IQR)] or mean [standard deviation (SD)] for continuous variables. Statistical analyses were performed with RStudio AGPL v3 Desktop Open Source Edition (RStudio Inc, Boston, MA).

## Results

Participants were *n* = 20 HCPs (19 females and 1 male; 8 nurses, 4 nurse practitioners, 4 respiratory therapists, and 4 neonatal fellows) who completed NRP-recertification within the last 24 months. The median [interquartile range (IQR)] number of months since participants completed their last NRP course was 6(1–10.5) years. The median(IQR) clinical neonatal experience was 10.5(3–17) years (Table 1).

**Table 1:** Descriptive data of neonatal HCP participants (*n* = 20).

**Characteristics (*****n*** **= 20)**	
Self-reported gender	19 female
	1 male
Current clinical position	8 nurses
	4 nurse practitioner
	4 respiratory therapist
	4 fellow
Neonatal experience	10.5(3–17) years [median (IQR)]
Time since NRP recertification	6(1–10.5) months [median (IQR)]
Board game experience	22.5(11–30) years [median (IQR)]
Enjoyment (5-point Likert scale)	4.1(0.6) [mean (SD)]

*Results are reported as mean [standard deviation (SD)] for normally distributed continuous data, and as median [interquartile range (IQR)] for non-normal continuous data*.

Overall *Open-answer test* performance was mean (SD) 8.6(2.1) out of 16 possible points (53%). Overall *Game Performance* was mean (SD) 29(3.2) out of 40 possible points (74%). Out of the 10 actions shared between the open-answer test and game scenario, performance on the *open-answer test* was mean (SD) 7.2(1.3) (72%) and *game performance* was mean (SD) 8.8(1.4) (88%). Also, the non-parametric Wilcoxon paired *t*-test revealed that the *game* score was significantly higher than the *open-answer test* score for the 10 shared actions (V = 17, *p* < 0.01). The paired samples *t*-test, using a cut-off of 65% as a passing score (12), revealed that 14/20 participants passed the *open-answer test* compared to 19/20 participants passed the *game* (*p* = 0.008).

There was no difference in performance on the task of attaching the pulse oximeter between the *open-answer test* and the *game performance* was *X*^2^ = 22.5 (*p* = 0.11), and the task of assessing breathing between the *open-answer test* and the *game performance* was *X*^2^ = 0.96 (*p* = 0.32). There was also no difference on tasks of heart rate assessment or initiating positive pressure ventilation between the *open-answer test* and the *game*. The task of stimulation was administered more frequently in the *game* compared to the *open-answer test* (*p* = 0.003).

All HCPs wrote the acronym for the six ventilation corrective steps (MR SOPA) on the *open-answer test*, while 18 out of the 20 HCPs correctly demonstrated all six ventilation corrective steps (MR SOPA) in the *game*. When further prompted to explain the steps that MR SOPA represents (Mask adjustment, Reposition airway, Suction mouth and nose, Open mouth, Pressure increase, and Alternate airway), 15 out of the 20 HCPs provided correct answers (e.g., O represents “Oxygen” instead of “Open mouth”).

When asked if participants enjoyed playing the game, five strongly agreed, twelve agreed, and three were neutral. Thus, 17 out of 20 (i.e., 85%) enjoyed playing the game. The number of years of overall game board experience was median(IQR) 22.5(11–30). Specifically, 75% of participants reported playing between 1–2 h of board games per week, whereas 25% of participants reported not playing board games at all during a typical week.

## Discussion

Each year, millions of newborns around the world will require help from HCPs to take their first breaths (13). To perform these life-saving interventions quickly and correctly, HCPs must master the knowledge and skills outlined in the neonatal resuscitation algorithm (4). However, neonatal resuscitation is a highly complex task, therefore deviations from the NRP algorithm are common. Retrospective observational studies have reported clinical error rates between 28 and 54%, depending on the complexity of the resuscitation (14, 15). To address this staggering gap and improve patient safety and health outcomes, frequent assessment of the HCPs' neonatal resuscitation competence is needed as a quality-improvement strategy (3).

Simulation is an effective method to train neonatal resuscitation and it has been incorporated during the NRP provider certification as a team-based exercise (16). Although simulation is often used to train HCPs' knowledge and skills, it remains an underutilized method to assess HCPs' clinical competence (7). This is because current approaches present barriers to the uptake of frequent simulation-based summative assessment in continuing neonatal resuscitation healthcare education (7).

Simulation is often unstructured, largely left at the discretion of the instructor, which may lead to conflicting evidence of its validity and reliability (7). In contrast, while traditional assessment formats (e.g., multiple-choice exams) provide a standardized alternative, these methods are often not relevant to the healthcare competency being tested (e.g., psychomotor skill) (6, 17). Moreover, summative assessments usually lack feedback or provide poorly designed feedback at the end, which may either demotivate HCPs from continuing health professional education (6, 17) or be received too late to be of use in the current learning context. Furthermore, resource constraints present a major barrier to frequent opportunities for high-fidelity simulation-based assessment (7, 8).

Serious games like RETAIN present an attractive alternative, with the potential to overcome the limitations of traditional summative assessment methods of clinical competence. The RETAIN board game provides a standardized approach to simulation, using evidence-based simulation scenarios and a structured scoring system for instructors to evaluate HCPs' performance (18). As a simulation-based serious game, RETAIN maintains its clinical relevance while meeting educational expectations as a summative assessment tool. Therefore, HCPs are provided with more meaningful feedback, as when players perform the correct steps of the neonatal resuscitation algorithm in the correct order, the infant's health improves and is successfully stabilized. Conversely, if inappropriate actions are taken, the infant's health deteriorates indicated by poor cardiorespiratory feedback. Lastly, RETAIN is accessible by HCPs from a variety of resources (e.g., no external resources or Internet connections are necessary) or backgrounds (i.e., the current work suggests that most participants are familiar with board games) and it can be used whenever and wherever is convenient (18), depending on the time available between neonatal resuscitation tasks to keep their NRP skills sharp.

Initially, RETAIN was developed for training neonatal resuscitation competence (18–20). However, the present study focuses on evaluating if the RETAIN board game can also be utilized as a summative assessment of HCPs' neonatal resuscitation competence. Summative assessments evaluate an individual at the end of a learning unit by generating a final report of their performance, as demonstrated on a particular task, in comparison to their peers or to an expected standard (6, 21). Therefore, we compared HCPs' performance on the RETAIN game to their performance on a traditional summative assessment method (i.e., a written open-answer test).

Participants were experienced neonatal HCPs from a tertiary perinatal care center, with a median experience of 10.5 years. Therefore, we expected participants to be competent at providing neonatal resuscitation. We observed that HCPs demonstrated improved performance on the RETAIN board game, compared to their performance on the open-answer summative assessment. Moreover, while only 14 out of 20 HCPs passed the open-answer assessment, 19 out of 20 HCP participants passed the game assessment (*p* = 0.008). Therefore, RETAIN may be a more telling assessment of actual neonatal resuscitation competence that may uncover learning aspects missed by more traditional NRP assessments.

The RETAIN board game seemed to uncover more of HCPs' knowledge of the neonatal resuscitation algorithm, compared to their responses on the open-answer summative assessment. For example, to assess knowledge of the ventilation corrective steps, the open-answer summative assessment elicited “1. MR. SOPA,” whereas the RETAIN game elicited: “1. Mask adjustment, Reposition airway, 2. Continue PPV and reassess, 3. Suction mouth and nose, Open mouth, 4. Continue PPV and reassess, 5. Pressure increase, 6. Continue PPV and reassess, 7. Alternate airway, 8. Intubation preparation, 9. Continue PPV and reassess.” While the open-answer test has the benefit of testing declarative knowledge rather than recognition, prompts during written simulation-based scenarios are often under-designed to elicit all the information sought by the assessor. It becomes difficult to adequately assess the learners' internal knowledge, as the learner must understand the question, then understand what the assessor is expecting, and then understand how to answer the question, all without feedback. In contrast, RETAIN offers opportunities for the assessor to provide meaningful feedback in real-time to prompt the learner for additional information of the important steps of neonatal resuscitation, similar to traditional simulation-based assessment (e.g., Objective Structured Clinical Examination). RETAIN potentially offers the benefits of a more standardized and robust simulation-based assessment method, while remaining resource-conservative similar to a traditional open-answer test.

Overall, players reported enjoying the game. This suggests that RETAIN might be a more attractive alternative for the assessment of HCPs.

Neonatal HCPs are expected to deliver high-quality care for some of the most vulnerable patients. Better health outcomes for newborn infants begin with better education for neonatal HCPs. Frequent and standardized summative assessment of neonatal resuscitation competence contributes to improved healthcare delivery and patient safety. The results from this study indicate that RETAIN may provide an alternative approach to assess how deeply learners understood the neonatal resuscitation algorithm. Furthermore, the RETAIN simulation-based board game provides an effective and engaging alternative to traditional summative assessment methods (e.g., multiple choice questionnaire, open-answer written test, etc.) (7). In addition, the RETAIN serious board game has the potential to overcome barriers (i.e., test-taking anxiety) of traditional summative assessment approaches. Other factors may influence participants' performance in the game, such as their mindset or their time elapsed until their last NRP course, as we have shown in our prior research using a video game (2). More research needs to be conducted to see if our prior results hold in the context of a board game.

Potential applications of RETAIN as a summative assessment include assessing preparedness for the NRP-provider course, assessing competency at the end of the recertification course, or for quality improvement strategies to continuously assess HCPs' competence in providing neonatal resuscitation.

## Limitations

Training with the RETAIN board game has been reported to improve knowledge retention of the neonatal resuscitation algorithm. However, we provided no guidance or help to participants. Therefore, the game functioned as a true summative assessment in this study. In addition, different interventions were required to successfully stabilize the infant across the two scenarios. While the two scenarios were of comparable difficulty considering the case history/extreme prematurity, comparison between the assessment methods may be limited to only the ten actions shared across both scenarios. Concomitantly, with a larger sample, a randomized assessment to either the open-answer test or the board game to be administered first will be considered to ensure no inadvertent learning happens during the traditional open-answer test.

In the open-answer test, participants needed to resuscitate, and stabilize an apneic 24-week premature infant. However, this scenario may need adaptation if used in future research, as we acknowledge that the practices of resuscitating premature infants may vary across cultures.

Another limitation was that, although players could see all the action cards while playing, potentially cuing their knowledge, similar conduits are also present during traditional simulation (i.e., the NRP algorithm flowchart posted at the bedside). However, this may have meant that the open-answer test measured declarative knowledge while the board game measured recognition of knowledge. Lastly, only 20 HCPs were recruited, and the limited number of data points precluded deeper analysis, such as between sub-groups analyses. This limitation stems from the time-intensive and effort-intensive administration and coding of the board game assessment and data, as well as from the limited number of HCP available to participate due to their busy work schedules. These limitations will be addressed in future RETAIN studies after collecting more data. Meanwhile, this is the first study to examine if the RETAIN board game can be used as a summative assessment tool.

## Conclusion

The current approach to assess neonatal resuscitation competence is infrequent, non-standardized, and potentially resource-intensive. The serious board game RETAIN provides an alternative toward objective and robust summative assessment of neonatal HCPs' resuscitation competence. Importantly, RETAIN offers an enjoyable, convenient, and low-maintenance learning environment, which may be motivating for continuing HCP education.

## Data Availability Statement

All datasets generated for this study are included in the article/Supplementary Material.

## Ethics Statement

The study was approved by the Human Research Ethics Board at the University of Alberta, Edmonton, Canada (Pro00085274), and written informed consent was obtained from the HCP participants prior to participation. The patients/participants provided their written informed consent to participate in this study.

## Author Contributions

GS, SG, and MC: conception, drafting of the manuscript, critical revision of the manuscript, and final approval of the manuscript.

### Conflict of Interest

GS has registered the RETAIN board game [Tech ID 2017083] and the RETAIN video game under Canadian copyright [Tech ID–2017086]. GS is an owner of RETAIN Labs Medical Inc., Edmonton, Canada (https://www.playretain.com) which is distributing the game. The remaining authors declare that the research was conducted in the absence of any commercial or financial relationships that could be construed as a potential conflict of interest.

## Abbreviations

HCP: Healthcare professional
NICU: Neonatal Intensive Care Unit
NRP: Neonatal Resuscitation Program
RETAIN: REsuscitation TrAINing for healthcare professionals.
